# Narratives of moral superiority in the context of war in Ukraine: Justifying pro‐Russian support through social creativity and moral disengagement

**DOI:** 10.1111/bjso.12878

**Published:** 2025-03-24

**Authors:** Ana‐Maria Bliuc, Daniela Muntele‐Hendreș

**Affiliations:** ^1^ Division of Psychology University of Dundee Dundee UK; ^2^ School of Social Sciences Western Sydney University Sydney New South Wales Australia; ^3^ Faculty of Psychology and Education Sciences University “A.I. Cuza” Iași Romania

**Keywords:** collective narratives, moral disengagement, social creativity strategies, violent intergroup conflict

## Abstract

The war in Ukraine has deepened ideological divides, particularly in neighbouring countries such as Romania and Moldova. This study examines how pro‐Russian supporters in these nations construct narratives to sustain moral superiority while justifying the invasion of Ukraine. Drawing on Social Identity Theory (SIT) and theoretical models of social creativity and moral disengagement, we analyse how a positive collective identity is maintained despite support for morally contentious actions. Through thematic analysis of social media content expressing pro‐Russian viewpoints, we identified strategies including reframing aggressive actions as morally justifiable, making favourable group comparisons, and emphasizing ingroup virtues while dehumanizing the outgroup. These approaches facilitate rationalization, mitigate cognitive dissonance, and preserve perceptions of moral superiority. Conspiracy theories about global powers manipulating the conflict further reinforce distrust in mainstream narratives and absolve Russia of responsibility. Our findings highlight how social identity mechanisms function to protect group identity, potentially intensifying ideological divisions and bolstering support for morally problematic positions. This research also provides insights into ways of combating misinformation and developing effective counter‐narratives in modern geopolitical conflicts.

## INTRODUCTION

The war in Ukraine has had far‐reaching consequences, deepening ideological divides across Europe, and particularly in neighbouring countries such as Romania and Moldova. In these contexts, public opinion is polarized, with narratives that either support or oppose Russia's actions being amplified by social media and misinformation (Bennett & Livingston, 2018; Bliuc & Chidley, 2022; Waisbord, 2018). Here we examine how pro‐Russian supporters in these nations construct and promote collective narratives that justify the war, focusing on the social psychological mechanisms enabling them to preserve perceived moral superiority of their ingroup. In conflict situations, public discourses play a crucial role in shaping public perceptions, often escalating support for violence (Bar‐Tal et al., 2014; McLaughlin, 2020). Societies involved in conflict frequently construct narratives that not only justify their actions but also strengthen the group's moral standing (Halperin & Gross, 2011). Our focus is on the pro‐Russian narratives emerging in countries which, even if they are not directly involved in the conflict, have been significantly affected by it. That is, we examine how pro‐Russian supporters in neighbouring Romania and Moldova construct and maintain collective narratives about the war in Ukraine. We argue that these narratives serve as expressions of social identity and employ specific strategies to maintain moral superiority despite supporting morally controversial actions.

Thus, drawing on the social identity approach (Tajfel & Turner, 1979; Turner et al., 1987) and concepts of moral disengagement (Bandura, 1990, 1999), and by investigating how these narratives function as part of a broader collective identity process, we seek to better understand how groups manage to maintain a positive moral (social) identity in morally ambiguous contexts. As such, our driving question is “how groups are able to maintain a positive social identity when they do something morally problematic?”, or in other words, what social identity management strategies are used when there is a misalignment between ingroup behaviour and the moral values consistent with the social identity. This broader inquiry can be divided into two specific research questions are: (RQ1) How do pro‐Russian supporters use social creativity strategies to maintain a positive group identity? and (RQ2) What moral disengagement mechanisms are used in pro‐Russian narratives about the war?

### Social identity and collective narratives

From a theoretical point of view, support for Pro‐Russian collective narratives can be understood as social identification with “opposing ideological camps”—that is, psychological groups formed around a shared interpretation of social reality (Bliuc et al., 2015, 2021; Bliuc, Betts, et al. 2024; Hogg, 2016). We argue that the endorsement of mutually exclusive collective narratives is underpinned by the interplay between (ideological or opinion‐based) group identity, social identity content, and collective narratives, as individuals define their self‐concept primarily through group membership, and these memberships are built around a shared interpretation of social reality (Hilton & Liu, 2017; Liu et al., 2014; Liu & Hilton, 2005; Tajfel & Turner, 1979; Turner et al., 1987). These narratives are not just passive reflections of reality, but they actively shape and reinforce the social identity content of the group (Bliuc & Chidley, 2022; Liu & Hilton, 2005)—that is, the shared values, beliefs, norms, and goals that define a group's collective identity (Livingstone & Haslam, 2007; Pehrson et al., 2009; Reicher, Cassidy, et al., 2006; Reicher, Spears, & Haslam, 2006). Collective narratives enable group members to establish their distinctiveness in the social context and, at the same time, help justify their positions on social and political issues and give meaning to their lives (Hammack, 2008, 2011). In polarized contexts, collective narratives tend to become mutually exclusive as psychological divides deepen, as for instance, in debates over climate change, immigration, or historical ethnic conflicts where opposing groups may frame the outgroup as fundamentally wrong or posing existential threats to the ingroup (Bar‐Tal et al., 2014; Bliuc et al., 2015; Hammack, 2008). These collective narratives not only describe but actively shape the group's identity, influencing both internal dynamics and relations with outgroups. Endorsing these narratives reflects a deeper process of social identification (and the internalization of social identity content), where group membership is tied to adopting its view of social reality. At the core of this identity are specific moral values that enhance group cohesion and distinction from the outgroup (Bliuc & Chidley, 2022).

In times of political upheaval, social unrest, polarization, or intergroup conflict, issues of morality and ethics tend to become highly prominent in public discourse (Van Leeuwen & Park, 2009). People find themselves questioning what is right and wrong and what their moral obligations are to themselves and others; so in these conditions, groups may end up endorsing morally flawed narratives (Bliuc, Hamilton, et al., 2024). For instance, in the context of the war in Ukraine, some studies suggest that supporting the pro‐Russian side involves defending morally corrupt positions (Khomyakov, 2023), whereas others highlight the influence of nationalist rhetoric, weaker political identities, and significant social media manipulation in shifting support for these positions (Geissler et al., 2022; Sokolova, 2023).

In the context of the war in Ukraine, we propose that there are two distinct and opposing narratives which have emerged. The first is a “pro‐Ukrainian” narrative, which presents a version of social reality where the war is viewed as highly traumatic, unjust, and unjustified, with the Russian military being cast as aggressors invading an independent nation. The second is a “pro‐Russian” narrative, which offers an alternative version of reality. In this narrative, the war against Ukraine is portrayed as legitimate and framed, for instance, as an effort to help the Ukrainian people by eliminating neo‐Nazi elements from their leadership and military. We argue that the endorsement of these mutually exclusive narratives (in online forums, for example) represents a proxy for identification with opposing ideological camps (Bliuc et al., 2015, 2021). As an ideological camp underpinned by a specific social identity, the pro‐Russian camp is characterized by identity content incorporating a specific shared set of beliefs and values (Bliuc, Betts, et al. 2024; Bliuc, Hamilton, et al., 2024; Livingstone & Haslam, 2007; Reicher, Cassidy, et al., 2006; Reicher, Spears, & Haslam, 2006). That is, as group members, pro‐Russian supporters are motivated to preserve their collective self‐worth, which they achieve by employing social identity management strategies to protect the group's moral integrity—strategies that are crucial for maintaining perceived moral superiority in the context of the war in Ukraine.

To summarize, our argument is that pro‐Russian online communication represents an expression of a specific social identity, driven by the group members' identification with an ideological camp driven by a pro‐Russian collective narrative (characterized by open or veiled support for Moscow's regime and its actions). Ideological camps, like other social groups, are based on norms that reflect their shared beliefs and values; thus, for the pro‐Russian camp, maintaining their status in the form of moral superiority represents a key group goal, central to their collective identity, but harder to achieve given the challenges of morally justifying acts of war. Next, we explore how group members can achieve these goals by employing specific strategies.

### Incorporating moral disengagement into social creativity strategies to preserve the ingroup's moral status

To understand how pro‐Russian supporters use rhetoric in attempts to maintain their moral group status, we turn to the social identity literature with a specific focus on strategies used by group members when their social identity is threatened. In particular, we refer to social creativity strategies and their potential to function to assert the group's moral standing by integrating specific moral disengagement mechanisms (Bandura, 1990, 1999, 2002). The use of these strategies allows group members to mitigate cognitive dissonance and achieve a sense of moral superiority, even when defending morally questionable positions and actions. We conclude this section by offering a framework for analysing pro‐Russian discourse, incorporating both social creativity and moral disengagement strategies.

The social identity theory (SIT, Tajfel & Turner, 1979; Turner et al., 1987) proposes that groups strive to maintain a positive self‐concept, often through adopting social creativity strategies that, while not directly attempting to achieve social change, are aimed at boosting group members' threatened social identity (Jetten et al., 2005; Van Bezouw et al., 2021). Social creativity strategies enable groups to maintain a favourable image despite controversial actions, serving as coping mechanisms for members to preserve a positive self‐concept and navigate challenging social situations.

When upward mobility to a higher‐status group is not an option or not preferred, these strategies become crucial. Rather than seeking individual status improvement by leaving the group, members employ social creativity to enhance their group's perceived status (Haslam, 2001). Social creativity strategies can be implemented by: (a) changing the context of intergroup comparison (e.g. using comparisons with lower‐status groups rather than higher‐status groups); (b) changing the dimension of comparison (e.g. “we might be poorer than X, but we are happier/more beautiful/more culturally rich”); or (c) changing the meaning of the group's identity content by reframing and redefining the identity to highlight its distinctive strengths. These strategies can be categorized as based on a manipulation of the comparison elements (changing the dimension of comparison and changing the group of comparison) or as based on a broader re‐definition of the meaning of group position and group membership (Reicher et al., 2012). In the context of this study, the second type of social creativity strategy, where social identity content is altered to sustain and enhance a positive group identity, is the most relevant.

In light of this, we propose that the social creativity framework can be applied to understand how groups attempt to maintain a positive (*moral*) social identity when there is a threat to their identity—in particular, that groups use specific social creativity strategies based on manipulations of social identity content in cases where upward social mobility may be possible, but not desired or applicable (as in the cases where group identities are based on conviction and ideology, rather than in social categories or other, more fixed, group memberships).

Such social creativity strategies which manipulate the content of a social identity are particularly relevant in contexts where a group's moral standing is threatened. When a group endorses actions perceived by others as morally questionable, maintaining a positive group identity becomes challenging. However, morality is not an objective or universally fixed construct; it is shaped and negotiated within the group's social and ideological framework. Using social creativity strategies, groups can reframe their actions as morally aligned with their values, enabling them to preserve a positive group identity while countering external critiques of their behaviour. It is in these circumstances that social creativity intersects with *moral disengagement* mechanisms. Moral disengagement refers to cognitive processes that allow individuals and groups to rationalize unethical behaviour (Bandura, 1990, 1999).

The need to justify one's attitudes and actions as morally acceptable is a deeply rooted aspect of human psychology, as moral transgressions threaten individuals' self‐concept and can lead to ethical dissonance (Bandura, 1999; Tsang, 2002). Violating moral standards causes ethical dissonance, threatening an individual's self‐worth (Ayal & Gino, 2012; Shalvi et al., 2015; Tsang, 2002). In response, people often engage in moral cleansing—symbolic actions that restore their sense of integrity (Dunning, 2007; Gollwitzer & Melzer, 2012; Sachdeva et al., 2009). For example, an environmentalist who buys a harmful product may volunteer for environmental causes or make more ethical purchases, reaffirming their values despite the initial transgression. Alternatively, when people support morally flawed positions that contradict their values, they may use moral disengagement strategies to restore their moral identity and cope with ethical dissonance (Bandura, 1990, 1999). These strategies help individuals distance themselves from the negative implications of their actions, rationalizing behaviours that conflict with their moral standards. This allows them to engage in otherwise immoral actions without experiencing significant dissonance or guilt.

Bandura's (1999) moral disengagement theory identifies cognitive mechanisms that individuals and groups use to rationalize unethical actions, allowing them to bypass moral self‐regulation and reduce cognitive dissonance. These mechanisms can be grouped into behavioural, agency, and victim‐focused strategies. Behavioural strategies include moral justification, where harmful actions are reframed as serving a noble purpose (e.g. violence as a means of preserving cultural traditions), and advantageous comparison, where one's actions are deemed less severe when compared to worse behaviours by others (e.g. justifying aggression by highlighting greater harm caused by another group). Agency‐focused strategies involve displacement of responsibility, where blame is shifted to authority figures or external circumstances (e.g. “It's not our choice; we are simply following orders”), and diffusion of responsibility, where accountability is diluted across a group. Victim‐focused strategies include dehumanization, which involves stripping victims of their humanity (e.g. portraying them as threats or subhuman), and attribution of blame, where victims are held responsible for their own suffering (e.g. suggesting they provoked aggression). These strategies allow individuals to rationalize morally questionable behaviours while maintaining a positive self‐concept (McAlister et al., 2006). In this study, these mechanisms are analysed as they are reflected in pro‐Russian narratives, providing understanding into how moral disengagement supports the justification of aggressive actions in a geopolitical conflict.

While traditionally seen as an individual cognitive mechanism, moral disengagement can also function at a group level (Newman et al., 2019). For instance, research shows that these strategies can become normalized within groups, reducing group‐wide self‐regulation (Huang & Yan, 2014). Though much of this research focuses on business ethics, studies have also examined collective moral disengagement among school children and adolescents (Gini et al., 2015; Pozzoli et al., 2012).

We extend this line of inquiry by exploring moral disengagement as a collective process used to protect a group's moral status in the context of war. For example, pro‐Russian supporters may reframe the invasion of Ukraine as defending traditional values, engaging in social creativity by shifting focus from aggression to cultural preservation. This reframing serves as moral justification, a key mechanism of moral disengagement, allowing members to disengage from ethical implications and reduce cognitive dissonance, at the same time sustaining a positive moral identity. We expect that, while social creativity maintains group identity, it often intersects with moral disengagement when groups seek to justify support for morally controversial actions, such as war.

### Current study

The war in Ukraine presents critical geopolitical and moral dilemmas. Russia's invasion of an independent nation raises critical ethical questions, prompting an analysis of narratives that sustain moral superiority amid such transgressions. This study examines the interplay of identity and morality, focusing on pro‐Russian narratives in Romania and Moldova, neighbouring countries indirectly impacted by the conflict. The controversy surrounding pro‐Russian political support in Romania and Moldova stems from the complex geopolitical dynamics in the region. In Moldova, the breakaway region of Transnistria, a Russian‐backed territory, has long been a source of tension, driving ideological divides that resonate in public opinion. Similarly, in Romania, proximity to Ukraine and its role as a NATO member have amplified debates on the legitimacy of Russian actions and their implications for national security. The war in Ukraine has further polarized these discussions, as pro‐Russian narratives often clash with the broader European condemnation of the invasion. These regional factors add layers of complexity to the social and political landscape, shaping the narratives analysed in this study.

We propose that pro‐Russian narratives are driven by two core motives: expressing a specific collective identity and maintaining a positive moral group identity. These collective narratives serve as strategic tools for preserving collective self‐worth, even when supporting actions that violate moral principles, including human rights abuses. Theoretically, we argue that these narratives underpin social creativity strategies incorporating moral disengagement, enabling group members to maintain or enhance their perceived moral status despite endorsing morally questionable actions.

While previous research has examined social creativity and moral disengagement as separate constructs, there is a clear gap in the understanding of how these frameworks intersect within emerging collective narratives in contemporary violent intergroup conflict. Our study seeks to address this gap by providing an integrated analysis of pro‐Russian narratives using both social creativity and moral disengagement theories. We focus on social media discourse from pro‐Russian supporters in Romania and Moldova, two culturally and geopolitically relevant contexts that have been significantly impacted by the war due to proximity, shared borders with Ukraine, and a large influx of refugees. Romania's status as a NATO member adds further complexity to the geopolitical dynamics in this region.

We expect that pro‐Russian supporters are likely to employ social creativity strategies to protect their group's moral standing while simultaneously asserting dominance over outgroups. However, given the considerable moral challenges inherent in defending pro‐war positions, we are interested in identifying how these strategies may incorporate moral disengagement mechanisms to justify the Russian invasion of Ukraine, while allowing supporters to maintain moral superiority. Specifically, we explore how individual‐level moral disengagement processes, such as moral justification and advantageous comparison, are integrated into group‐based social creativity strategies to reinforce a positive moral identity. We expect that the use of these strategies would help sustain the group's moral status within a broader hierarchy of values, despite the difficulty of justifying war and the severe moral violations reported against civilians.

To sum up, our study seeks to determine how moral disengagement strategies are integrated within a broader social creativity framework to maintain and assert (moral) group superiority. At the same time, we seek to determine whether strategies other than moral disengagement are used to justify pro‐Russian positions.

## METHOD

This study employs thematic analysis, a qualitative research approach that enables the systematic identification, organization, and interpretation of patterns or themes within textual data (Braun & Clarke, 2006). Thematic analysis was chosen due to its flexibility in capturing the nuanced and context‐dependent nature of discursive constructions in pro‐Russian narratives. By focusing on both deductive and inductive coding, this approach allowed us to explore predefined theoretical constructs (e.g. moral disengagement strategies) while remaining open to emergent themes in the data. This choice aligns with the study's aim to examine how moral and identity‐based justifications are articulated in social media discourse, providing an analytic lens that is grounded in psychological and rhetorical traditions.

The extracts presented in the findings section were carefully selected to provide illustrative examples of the broader dataset. These extracts were chosen because they exemplify the key themes identified during the thematic analysis and reflect the diversity and depth of the pro‐Russian narratives captured in our study. While they do not encompass the entirety of the data, they provide an accurate snapshot of the database and are representative of the recurring patterns and strategies observed across the dataset. This approach ensures that the findings are both grounded in the data and accessible to readers.

### Data collection

#### Data sources

The data were collected from publicly available social media platforms and online forums, which are known to sometimes include automated accounts (bots). While this is a potential limitation, our analysis focused on qualitative interpretations of discursive content, emphasizing patterns and strategies that are indicative of human reasoning and social identity processes. Posts and comments with characteristics typical of automated activity, such as repetitive or incoherent language, were excluded during the data‐cleaning process. Additionally, the focus on content with higher engagement levels (e.g. likes, replies) likely reduced the influence of bots, as these interactions tend to reflect genuine human discourse. The database for this study was created using posts and comments from publicly accessible social media platforms, including Facebook, Instagram, and region‐specific blogs commonly used in Romania and Moldova (e.g. “Blog Ortodox”). These platforms were chosen for their significance in hosting political discourse and pro‐Russian sentiment. The dataset comprises 230 text entries, including posts and comments, collected over a six‐month period from accounts explicitly expressing pro‐Russian views related to the Ukrainian conflict.

Data were collected through purposive sampling, prioritizing content with high engagement levels (e.g. likes, shares, replies) as indicators of relevance and influence within online communities. Each entry was annotated with details about its type (post or comment), platform of origin, and thematic focus. All entries were in the Romanian language and were translated into English by the authors, who are native Romanian speakers. Ethical considerations were integral to the data collection process. Only publicly accessible content was used, ensuring no private or confidential information was included. The data collection adhered to the ethical guidelines of the British Psychological Society and received approval from the Ethics Committee at the university where the study was conducted.

#### Sampling strategy

A purposive sampling strategy was employed to select posts and comments that explicitly expressed pro‐Russian viewpoints related to the Ukrainian conflict. This strategy ensured that the collected data were rich in content relevant to the research question. A total of 230 social media posts and comments were selected for analysis. These posts were selected based on their engagement levels (e.g. the number of likes, shares, and comments), which served as indicators of their influence and relevance in the online community. The sampling strategy exclusively targeted social media posts and comments written in Romanian, reflecting the study's regional focus on Romania and Moldova, where Romanian predominates in online political discourse. Posts in other languages (i.e. Russian) were excluded to maintain consistency and ensure linguistic coherence in the analysis.

#### Positionality statement

We disclose our positionality to ensure transparency regarding the perspectives shaping this research. We unequivocally condemn the war in Ukraine, recognizing it as a humanitarian tragedy and a violation of sovereignty. This stance reflects our commitment to democratic principles and human rights. As Romanian nationals, we are acutely aware of the conflict's regional consequences, particularly its impact on public opinion and geopolitical stability. Our proximity offers contextual insight but also points out to our personal connection to the war's implications for Romania's democratic institutions and regional security. This dual perspective—as researchers and individuals influenced by Romania's socio‐political environment—has informed our analysis of the narratives surrounding the conflict. We approach this work with scholarly rigour and reflexivity, acknowledging the influence of our cultural and national identity on our interpretations. This statement reaffirms our dedication to impartiality and adherence to the ethical standards of our discipline.

#### Ethical considerations

Given the sensitive nature of the topic and the potential risks to individuals expressing controversial opinions, ethical considerations are important. All collected data were non‐identifiable, and only publicly available data were used, ensuring that no private communications were accessed. The ethical protocol for this study was reviewed and approved by the Ethics Committee at [University Name to be added after peer‐review] and adheres to the ethical guidelines outlined by the British Psychological Society.

### Data analysis

#### Analytical approach

The data were analysed using a thematic analysis approach, as outlined by Braun and Clarke (2006, 2019). The analysis followed both deductive and inductive processes to ensure in‐depth understanding of the data. Our analysis was organized according to the following stages (based on the phases recommended in thematic analysis):

1. To familiarize oneself with the data, all text (posts and comments) were read; this initial read was followed by a more in‐depth examination of the data.
2. Next, keywords and codes within the text as a whole were identified (an inductive approach).
3. We examined the codes and ordered them according to their degree of fit within the pre‐determined categories (a deductive approach); for the codes which did not fit into these categories, we organized them into new categories (emerging from the data).
4. Finally, we organized the categories within themes and selected illustrative quotes for each theme.

#### Deductive coding

The initial coding framework was developed deductively based on existing theoretical concepts from social identity theory and moral disengagement theory. Categories such as “moral justification”, “euphemistic language”, “advantageous comparison”, and “dehumanisation of the outgroup” were predefined, based on Bandura's (1999) moral disengagement mechanisms and Haslam's (2001) description of social creativity strategies.

#### Inductive coding

In parallel, an inductive approach was used to allow the emergence of themes not captured by the initial coding framework. This involved reading and re‐reading the data to identify patterns or themes that were not anticipated initially. This approach ensured that the analysis was grounded in the data and captured the nuances of the narratives.

#### Coding process

The coding process involved two stages. First, the researchers independently coded a subset of the data to develop a preliminary coding framework. Any discrepancies in coding were discussed and resolved through consensus, and the coding framework was refined accordingly. In the second stage, the remaining data were coded using the final framework.

#### Theme development

After coding, the codes were grouped into broader themes that capture the underlying narrative strategies. The themes were reviewed and refined through an iterative process to ensure that they accurately reflected the data and theoretical frameworks. Each theme was supported by representative quotes that were translated from Romanian to English by native speakers, maintaining the original meaning and context.

#### Reflexivity

The research team sought to remain reflexive throughout the study, particularly in acknowledging potential biases stemming from their cultural and political perspectives. Regular reflexive discussions were held to ensure that these biases were recognized and addressed in the interpretation of the data. While these findings are specific to the context of pro‐Russian narratives in Romania and Moldova, theoretical insights into the integration of social creativity and moral disengagement have broader applicability. The detailed description of the research context and methodology allows for the transferability of the findings to similar contexts, such as other geopolitical conflicts where moral justification plays a critical role.

## FINDINGS

First, through the deductive approach, our thematic analysis identified four primary themes in the pro‐Russian narratives: “Reframing actions”, “Comparing ingroup and outgroup behaviours”, “Highlighting ingroup virtues versus dehumanising the outgroup”, and “Nationalism and ingroup superiority: self‐preservation as a driver”. Each theme represents a distinct social creativity strategy used in attempts to maintain moral superiority and justify actions through moral disengagement. These themes suggest that social creativity is predominantly used to change the meaning of the ingroup within the context of the conflict (i.e. to reframe the content of the social identity in a way that would preserve or enhance the moral status of the group). Social creativity is used to a lesser extent to frame a group as morally superior by changing the comparison group and the dimension of comparison. Second, when using an inductive approach to our data analysis, we identified a broader theme that relied heavily on conspiracy theories to “uncover the truth” about the ongoing war. This theme is underpinned by the subthemes of mistrust in official or mainstream news sources.

### Reframing actions to misrepresent harm

A prominent theme within pro‐Russian narratives is reframing aggressive actions to portray them as morally justified. This strategy involves shifting the focus away from the harm inflicted on the Ukrainian population, instead emphasizing purported benefits such as the defence of traditional values or the preservation of cultural practices. For instance, narratives often recast the invasion as a necessary intervention to protect Eastern European religious and cultural heritage from perceived Western encroachment, thus legitimizing the violence through a moralized lens. One common tactic is to recast events of the last 8 years in Ukraine by vilifying its leadership. For instance, they argue that:

*Extract 1*. Framing aggressive actions as culturally justified:

The truth about what happened in Ukraine in the last 8 years will show that they had a leadership of traitors who mocked their citizens, including those in the Donbas regions where many civilians and children were killed by Ukrainians. You all kept silent, hypocrites.

This type of narrative minimizes or misrepresents the harm caused, as exemplified in statements like “It is not as bad as it looks”, which attempts to shift focus from the suffering of the Ukrainian people to alleged gains or benefits, thereby changing the dimension of comparison from losses to supposed gains.

Additionally, responsibility is often displaced onto other entities. For instance, some online contributors argue “The UN and EU are responsible rather than Russia”, or deflect to unrelated issues, but important issues, asking “What about Romanian orphans and poor people?” In these cases, the focus is diverted from the harm caused by war to broader presumptions about the causes of war, sometime also invoking conspiracy theories. These narratives suggest that the conflict is driven by the interests of the European Union and the United Nations. One example of this is the claim shown in the extract below.

*Extract 2*. Mis‐attributing the sources of conflict:

Everything is a fight over/for resources; with the aid of the United States, they want to rip/destroy Russia so they can continue to live happily on Russian resources. As long as the globalising capitalists lead the world, there are going to be constant wars.

Here, the argument extends beyond Ukraine, placing responsibility on entities like the UN and EU, and suggesting that their influence is pervasive across all global conflicts.

More extreme versions of this argument portray the Russian president as a potential saviour of traditional Eastern European values against the perceived corruption of the West. For example, some argue “Putin knows where Romania is; if he wants to bomb it, he will, but he doesn't want to. If he does, it will be with an objective, to save us from NATO and the EU, so we can live freely” (*Extract 3*. Putin as saviour).

This type of narrative often involves distorting social reality or diffusing responsibility, as seen in arguments such as:

*Extract 4*. Diffusion of blame:

Who is to blame? General context? Western threats to traditional Eastern European values? Stop only blaming V.P. (Vladimir Putin). Others are responsible for this disaster! Arrogance and evil are also in other presidents who hold responsibilities. COVID destroyed families, led to death…we all suffered…they will make us all slaves.

Overall, in pro‐Russian narratives within this thematic category, reframing aggressive actions is key to maintaining moral superiority. By portraying the invasion of Ukraine as a defence of traditional Eastern European values, supporters use social creativity to shift the narrative from aggression to cultural preservation. This reframing not only enhances the group's moral status but also enables moral disengagement, justifying actions that would otherwise be condemned. It shifts focus away from the immediate suffering caused by the invasion, emphasizing a supposed higher moral purpose, allowing supporters to back the war without experiencing significant moral conflict.

### Comparing ingroup and outgroup behaviours (using advantageous comparison)

While strategies such as advantageous comparison are not unique to pro‐Russian narratives and align with broader social identity theory (SIT) processes, the pro‐Russian position in this context is distinguished by its use of these strategies to defend actions widely perceived as morally transgressive. Specifically, the pro‐Russian narratives examined in this study employ mechanisms of moral disengagement to justify aggressive actions, including the invasion of Ukraine and harm inflicted on civilians. In contrast, pro‐Western narratives, although they may similarly utilize strategies like moral justification or advantageous comparison to uphold moral superiority, generally do not involve defending direct violations of international law or acts of aggression. Instead, these narratives typically frame support for Ukraine as consistent with democratic values and humanitarian principles. This distinction highlights the differing moral and geopolitical implications of the two positions, with the pro‐Russian stance justifying actions deemed ethically contentious. By framing pro‐Russian narratives within this context, we show how social identity processes and moral disengagement mechanisms uniquely intersect to sustain support for problematic positions.

In political conflicts, advantageous comparison is frequently used to rationalize violent behaviour by contrasting it with the actions of more extreme or threatening groups. This strategy reduces the perceived severity of one's own actions and justifies them as less morally objectionable by comparison (Bar‐Tal, 1990; Tajfel & Turner, 1979). By using this mechanism, groups can reframe their behaviour in ways that align with their moral identity, even in the context of morally contentious actions. By focusing on these comparisons, individuals justify or excuse actions that may otherwise be considered morally wrong or unjustifiable. However, this can further polarize groups and escalate conflict, as each side competes in a “race to the bottom” of increasingly extreme and morally questionable behaviour. This technique is used here to improve the pro‐Russian group's image while downplaying their own actions as for example, when one online user asks: “If I am a war criminal, what are the American presidents from Nixon onwards?” (*Extract 5*. Rhetorical double standards).

In our analysis, this strategy was also evident when the focus shifted from the conflict between Russians and Ukrainians to Romanians and Ukrainians. By changing the comparison group, the historical and geopolitical contexts of the conflict were altered. This is illustrated in the following quote:

*Extract 6*. The “Plight” of oppressed Romanians:

Why don't you cry for the Romanians oppressed by the Ukrainian nationalists? They are our brothers. Why don't you ask for the Ukrainian president to give them rights? Weak minds…

Another frequently used comparison outgroup is “Americans”, where the argument shifts to how much worse Americans behaved in similar situations in the past. For example, the following excerpt changes both the comparison group and historical context:

*Extract 7*. Bringing up forgotten victims.

In Vietnam, the American army exterminated 3 million civilians and almost 2 million soldiers… this is beside the fact that they chemically burned the forests and mined all the agricultural fields, so that even now, country folk die on these fields, and children get injured (disfigured…). I would like to see now how many avatars will use the Vietnamese flag?

We found that these strategies often intermingle. For example, a shift in the comparison group could also lead to a shift in the ascription of blame. That is, when arguing that the Ukrainian government—or those allegedly controlling it—had the power to stop the conflict, but instead chose to antagonize Russia, the narrative is framed as follows:

*Extract 8*. Zelensky: puppet or patriot?

I agree that Zelensky, if he really acts in the interest of his people, should give up. But I don't believe that this depends on him. He is a puppet in the hands of the masters from the USA. And now he stabbed his own brain.

Similarly, within this theme, we see examples of the actions of the Russian military being contrasted with those of Western leaders or Ukrainian forces. This tactic allows the ingroup to appear morally superior by minimizing the perceived severity of their own actions, such as the military invasion of Ukraine or reported harm to civilians. For example, pro‐Russian narratives in the dataset frequently reframed these actions as defensive measures or acts of cultural preservation, downplaying their destructive impact. While not all of these examples are presented in the extracts, they are consistently observed in the dataset, illustrating how moral disengagement operates to rationalize aggression. That is, by comparing Russia's military interventions with the historical actions of the United States or NATO, these narratives downplay Russia's aggression, framing it as comparatively less harmful or even justifiable. This process serves not only to elevate the ingroup's moral status, but also to shift the focus from Russia's actions to those of other groups. Thus, in these cases, moral disengagement is reinforced through a narrative that normalizes or excuses unethical behaviour by contextualizing it within a broader, supposedly more brutal, historical framework.

### Highlighting ingroup virtues vs. dehumanizing the outgroup

Pro‐Russian narratives tend to also highlight the unique virtues and positive characteristics of the ingroup, contrasting these with the dehumanization of the outgroup. This dual approach serves to both reinforce the moral superiority of the ingroup and justify unethical actions against the outgroup. One common theme in these narratives was the emphasis on the ingroup's commitment to peace, determination, and moral integrity. For example, the following excerpt suggests a deep connection to land, heritage, and family, portraying these as inherent virtues of the ingroup.

*Extract 9*. Unity and resilience beyond the Prut:

We Romanians from Chișinău and Moldova, and you from beyond the Prut (referring to regions in Romania located on the western side of the Prut River, which forms part of the border between Romania and Moldova) regardless of what happens, war, or crisis of any kind, must remain on our lands, die on our lands with icons in our hands, and with the hope that we will die next to the graves and houses where we were born, where we grew up, and where we founded our families.

Interestingly, this quote also shows the framing of the ingroup as representative of a the broader (superordinate) national identity category, referring to the Romanian national identity, which is invoked in these narratives to emphasize unity, shared cultural heritage, and moral distinctiveness in contrast to other groups, thus attempting to present the ingroup as more prototypical and normative compared to outgroups, a technique that, according to the ingroup projection model (Wenzel et al., 2007, 2016), can contribute to increase negative attitudes and hostility towards outgroups. In addition, this narrative not only highlights the perceived moral integrity and resilience of the ingroup, but also invokes a sense of sacred duty and honour tied to the land and tradition. The imagery of dying with icons in hand reinforces a sense of spiritual righteousness, further solidifying the moral high ground attempted by the ingroup.

Conversely, the outgroup is often dehumanized, depicted as less than human, which serves to rationalize and justify any unethical actions taken against them. For instance, this is evident in the portrayal of the “Azov” fighters, where dehumanization is explicit: “Those Azov fighters are scoundrels; they should be shot; none should be captured alive” (*Extract 10*. Dehumanizing the Azov fighters). The reference to “Azov fighters” likely pertains to the Azov Brigade, a Ukrainian military unit that has historically been associated with the controversial use of Nazi symbology. Although this is largely considered an isolated case, it has been exploited by Russian propaganda to frame Ukraine as being “overrun by Nazis”, a central element in justifying the war. This context highlights how the dehumanization of the Azov fighters in pro‐Russian narratives is tied to broader anti‐Nazi discourse, which has been hijacked to rationalize aggression against Ukraine. By portraying the Azov fighters as emblematic of Ukraine's leadership, these narratives amplify their justification for violence while dehumanizing a specific subset of the Ukrainian population.

Such dehumanization facilitates the moral disengagement necessary to endorse or condone strong violence against the outgroup. By reducing the outgroup to mere “scoundrels” unworthy of life, the narrative strips them of humanity, making it easier to justify extreme measures, such as executing them without the possibility of capture.

The juxtaposition of highlighting ingroup virtues with the dehumanization of the outgroup creates a stark moral dichotomy, in which the ingroup is seen as inherently good and just, while the outgroup is depicted as irredeemably evil. This “black‐and‐white” framing seems to be used not only to reinforce group cohesion but also to escalate the potential for conflict, as it reduces the complexity of the situation to a simplistic battle between good and evil. The claim about escalating the conflict refers to the rhetorical and psychological effects of dehumanizing language within pro‐Russian narratives. By portraying outgroups, such as the Azov fighters, as irredeemably evil or subhuman, these narratives contribute to growing intergroup hostility. This, in turn, reinforces support for aggression and undermines efforts towards de‐escalation or resolution, as such framing normalizes violence and fosters a zero‐sum perception of the conflict.

This analysis highlights how moral disengagement strategies can manifest as discursive practices grounded in social interactions, demonstrating the dialogic complexity of these mechanisms. For example, narratives that reframed aggression as cultural preservation were often accompanied by explicit appeals to shared group values, such as tradition and heritage. These articulations function not only to justify actions, but also to reinforce a cohesive group identity. One participant argued that “the invasion is necessary to protect Eastern European heritage from Western corruption” (*Extract 11*. Necessary evils), thus presenting violence as a moral duty. Similarly, moral justification often operated alongside dehumanization, with statements like “those Azov fighters are scoundrels; (…) none should be captured alive” (see *Extract 10*), rationalizing extreme actions by stripping the outgroup of humanity. These examples are a good illustration of the dual role of discourse: it rationalizes morally contentious actions while embedding these rationalizations within social narratives that preserve ingroup identity.

### Nationalism and ingroup superiority: Self‐preservation as a motive

Within this theme, pro‐Russian narratives often employ moral justification by prioritizing nationalist values, which in turn are used to legitimize support for war. This approach frames the defence of national identity and sovereignty as a paramount duty, even if it involves supporting or engaging in conflict. A clear example of this is seen in the following excerpt, in which the poster justifies their stance by invoking the threat of war due to perceived provocations:

*Extract 12*. Patriotism and the perils of provocation:

We want peace and don't want to be visited by Putin because of the Ukrainian flags you are illegally displaying on state institutions and public spaces, violating law 75, article 7. Romania is the closest NATO flank, and we will be the first Russian target. If you want to fight with Russians, go to Ukraine!! Don't give them reasons to come for us! If we will be attacked, you with UA in your profile, who forgot our tri‐colour flag, will be the first to leave the country.

This statement reflects a form of moral justification in which the protection of the nation and its values is positioned above all else. This argument implies that displaying Ukrainian symbols in Romania is not only illegal but also dangerous, as it could provoke Russian aggression. In this example, the concern of the poster is framed as a defence of peace and Romanian sovereignty, suggesting that those who support Ukraine are not only risking the safety of the country, but are also betraying national values.

Further reinforcing this narrative, another quote expresses a preference for Russia over the United States while claiming a pro‐Romanian stance: “Some clarifications. About the war in Ukraine. I am not pro‐Russian, I am pro‐Romanian. But between Russia and the US, I prefer Russia. (…)” (*Extract 13*. Romanian patriotism above all). This statement reveals a more complex moral positioning where the poster distances themselves from outright support for Russia but, nonetheless, prefers it over the United States, based on a perceived alignment with national interests and shared cultural heritage (i.e. Christian orthodoxy in particular). This preference is framed in the extract as a pragmatic alignment with Russia rather than the West, suggesting a perceived benefit to national interests. However, while shared cultural heritage (e.g. religion, historical ties) may play a role in shaping these perceptions, further evidence from the dataset would be required to substantiate this specific claim.

Finally, the narrative extends blame to Western institutions, reinforcing the idea that external forces, rather than Russia, are responsible for the ongoing conflict:

*Extract 14*. Blame, lies, and manipulation:

EU and NATO are to blame for all that is happening!!! People watch news too much and believe all the lies!!! Brainwashing, global manipulation!

Here, the poster attempts to shift responsibility for the conflict to the EU and NATO, accusing them of manipulation and deceit. This serves as another form of moral justification, where the support for war or for Russia is rationalized as a defence against Western aggression and manipulation, rather than an act of aggression on the part of Russia.

In summary, these narratives use nationalist values as a moral framework to justify support for war or for Russia's actions, framing these stances as necessary for protecting national identity and sovereignty. This type of moral justification not only legitimizes support for war but also serves to rally individuals around a common nationalistic cause, possibly at the expense of international cooperation or peace.

### Conspiracy theories about the “truth” of what happens in Ukraine

Conspiracy theories are narratives that attribute significant events or actions to covert and often malevolent intentions of powerful entities, frequently in the absence of definitive evidence (Douglas et al., 2005, 2019). These theories typically rely on distrust of official accounts, patterns of secrecy, and the framing of events as deliberate manipulations for hidden agendas. In the context of this study, the pro‐Russian narratives identified as conspiratorial share several hallmark features, including scepticism towards mainstream media, accusations of global manipulation by Western powers, and the portrayal of Russia as a victim or saviour against alleged conspiracies orchestrated by entities such as NATO or the EU. For instance, the extracts in this section highlight how conspiracy perspectives are constructed through selective interpretation of events, such as the framing of the Odessa fire as evidence of Ukrainian fascism or the characterization of financial aid to Ukraine as part of a fabricated global scheme. These elements position the narratives within a conspiratorial framework that undermines alternative viewpoints and justifies pro‐Russian stances.

*Extract 15*. Reframing aggression:

The truest truth about the crimes of Ukrainian modern neo‐fascists. They had weapons and power and imagined that they were super‐humans. They robbed and killed ordinary people. And Putin brought the army and saved Ukraine from Nazis. But global mass media deceives the whole world and washes people's brains that Putin and Russia are the aggressors when in fact Russia is saving Ukraine from the fascists and thieves from the Ukrainian government. Second, on the 2nd of May 2014, Ukrainian fascists burnt people in Odessa.

The framing of the Odessa fires on 2 May 2014, in pro‐Russian narratives exemplifies selective descriptions of historical events to support a conspiratorial perspective. These narratives often highlight the tragic deaths of pro‐Russian protesters in the fire, portraying them as victims of Ukrainian fascism. However, this framing omits the broader context of the political unrest and tensions that characterized the situation. The events in Odessa occurred amid escalating conflict between pro‐Ukrainian and pro‐Russian factions, with violence erupting from clashes between both sides. By focusing exclusively on the suffering of pro‐Russian protesters while neglecting the broader dynamics of the unrest, these narratives simplify a complex and multifaceted event to reinforce their ideological position.

This selective emphasis indicates how pro‐Russian narratives use moral and emotional appeals to amplify distrust and justify their stance, often at the expense of a more nuanced understanding of historical events. This narrative is interesting because it inverts the widely accepted view of Russia as the aggressor, positioning Russia as the saviour of Ukraine, purportedly rescuing the country from a corrupt, fascist regime. The mention of the Odessa fire on 2 May 2014 is used to reinforce this alternate reality, emphasizing a selective historical event to validate the broader conspiracy.

Another angle of these conspiracy theories shifts the focus to financial resources pledged to Ukraine by its allies. This narrative suggests that the entire situation is a fabricated scheme designed to extract financial gains rather than a genuine response to the humanitarian crisis. This perspective is articulated in the following statement:

*Extract 16*. “Who pays the price?”

This is just theatre so aid is sent by all! Now Ukraine is collecting billions of Euros! Those who are stupid to believe them pay for the price rise of electricity and gas. Food and everything. Those who have will continue having while the poor die because they are stupid. The pandemic for those stupid starts again! Some are dying from poverty while others make movies to become rich overnight! I am not believing anything anymore! They should leave us with our problems!

Here, the narrative dismisses the war as mere performance designed to exploit global sympathies and gain financial support, while ordinary people are constructed as suffering due to rising costs of living. This framing taps into existing frustrations among pro‐Russian supporters and segments of the population who feel economically disenfranchised or sceptical of Western involvement in the region. By emphasizing economic hardships such as rising costs of living and portraying them as a consequence of supporting Ukraine, these narratives resonate with individuals who perceive themselves as bearing the brunt of geopolitical decisions made by Western powers. Similarly, there are expressions of scepticism towards Western support for Ukraine, reflecting distrust in the real motives behind Western support for Ukraine, with suggestions that this could be a strategy to weaken Russia or gain geopolitical benefits, as illustrated by this post:

*Extract 17*. Foreign demands always prevail:

America demands, the European Union demands… The bootlicking leaders give, steal, take… Isn't it time for the people to demand as well?

A further extension of these conspiracy theories draws parallels with historical events, such as Romania's involvement in World War II. The narrative warns that Romania is once again being manipulated by external powers, this time with Zelensky playing the role of a puppet.

*Extract 18*. “History repeating”:

Romania had fallen into the Germans' trap led by Hitler who was pushed from behind by the USA; now there is the same situation, but the actor is Zelensky. I don't believe that Romanians would be tricked again, but if we are, it means that we deserve our fate as a colony or whatever you think we still are for Europe.

This statement exploits historical memory, equating past and present events to suggest that Romania is being used as a pawn in a larger geopolitical game, just as it allegedly was during World War II. It plays on nationalist sentiments and fears of losing sovereignty, painting a picture of Romania as a colony of Western powers if it continues to support Ukraine.

Not surprisingly, within this theme, one key subtheme is *distrust in official war narratives*, reflecting the scepticism of many online posters towards information presented in mainstream media and by governments about the conflict. Many seem to believe that the public is presented with distorted or incomplete versions of events. For example, as one poster says, “I am not neutral and balanced regarding Ukraine. I believe Ukraine is a state fabricated by the Soviets, now led by a nauseating cocktail of neo‐Nazi nationalists and prostitutes manipulated by the West” (*Extract 19*. Western manipulation).

Overall, the use of conspiracy theories seems to serve to undermine the mainstream narrative of the conflict in Ukraine, instead promoting a view that absolves Russia of wrongdoing while casting doubt on the legitimacy and morality of Ukraine and its allies. By framing the situation as a global conspiracy, these narratives seek to delegitimize any support for Ukraine, fostering scepticism and mistrust among those who are susceptible to such claims.

## DISCUSSION

We begin our discussion by foregrounding the specific geopolitical and cultural context of Romania and Moldova, which shapes the pro‐Russian narratives analysed in this study. The historical ties to Russia, the ongoing influence of the Transnistrian conflict, and the proximity to the Ukrainian war create a unique backdrop for understanding how moral disengagement and identity strategies operate in this region. This context is particularly important for interpreting the narratives' reliance on conspiratorial framing, advantageous comparison, and moral justification, which resonate with local historical and socio‐political dynamics.

As Billig and Marinho (2017) argue, the strength of qualitative research lies in its capacity to use specific, contextually rich examples to generate broader theoretical insights. In this spirit, while our findings are based on the specificities of pro‐Russian narratives in Romania and Moldova, they open the door for broader observations about how moral disengagement strategies and social identity processes intersect in polarized geopolitical contexts. These broader implications are exploratory, providing a basis for future research that might test their applicability in other regions or conflicts.

We propose here that the theoretical integration of social creativity strategies with moral disengagement elements is central to understanding how groups maintain their moral superiority in morally challenging contexts. While social creativity strategies shift the basis of moral comparison, moral disengagement mechanisms justify the group's support for morally problematic actions. These processes work in tandem, as groups reframe harmful actions in a more favourable moral light while disengaging from the ethical implications of these behaviours. Our analysis shows how pro‐Russian group narratives systematically employ a social creativity approach that incorporates moral disengagement strategies to sustain a sense of moral superiority in the face of widespread opposing public views. By reframing aggressive actions as morally defensible, drawing advantageous comparisons with other groups, and simultaneously highlighting the ingroup's virtues while dehumanizing the outgroup, these narratives enable pro‐Russian supporters to rationalize and uphold their stance.

Our analysis examines how individuals construct and defend pro‐Russian positions as morally desirable within their social and ideological context, even when these positions may face external critique. This does not suggest that individuals act against their own moral beliefs; rather, the narratives illustrate a process of moral justification and identity preservation that aligns their actions with their internal moral standards. Although outsiders may perceive these positions as morally problematic, we argue that the individuals in the study employ strategies to present their stance as morally defensible, effectively challenging alternative perspectives. This highlights the role of moral disengagement mechanisms, not in rejecting morality, but in reconciling contested actions with the individuals' own coherent moral framework. Social identity theory suggests that groups maintain a positive social identity by engaging in social creativity, which involves altering comparison dimensions to favour the ingroup (Tajfel & Turner, 1979). In the context of pro‐Russian narratives, *reframing actions* is a clear example of this, where violent actions, often against civilians, are reinterpreted as morally justifiable, protecting vulnerable populations or defending traditional values. This reframing shifts the narrative away from the harm caused and towards a perspective that presents these actions as acts of defence or moral righteousness, consistent with social identity theory's predictions about group behaviour in conflict situations.

Our analysis demonstrates that pro‐Russian group narratives systematically intertwine social creativity and moral disengagement strategies in innovative ways as means to sustain a sense of moral superiority in the context of war. By reframing violent actions as morally justified and drawing favourable comparisons against Western powers, portraying Russian actions as morally superior or less harmful by highlighting perceived failings or more egregious actions by Western entities. Emphasizing ingroup virtues and dehumanizing the outgroup further reinforce these strategies, enabling members to rationalize and support morally questionable actions while preserving their positive social identity. This approach illustrates how the need for a positive collective identity drives moral disengagement, leading to the justification and perpetuation of conflict.

In addition to these cognitive strategies, narratives also focus on highlighting ingroup virtues. This tactic aligns with research on ingroup favouritism (Brewer, 1999), in which groups emphasize their positive qualities to enhance group cohesion and self‐esteem. In pro‐Russian narratives, qualities such as steadfastness, moral integrity, and a deep connection to heritage and land are highlighted, thereby constructing an image of the ingroup as inherently virtuous and resilient. This glorification of the ingroup serves not only to reinforce group cohesion but also to justify the group's stance in the conflict.

Conversely, narratives often dehumanize the outgroup, a process that is critical to moral disengagement. Dehumanization, as explored by Haslam (2006), involves perceiving others as less than human, which facilitates the justification of unethical behaviour towards them. In pro‐Russian narratives, outgroup members are frequently portrayed as “scoundrels” or “neo‐fascists” stripping them of their humanity and making violence against them seem benign. This process of dehumanization is a powerful tool for moral disengagement, allowing the ingroup to rationalize extreme actions and frame them as morally acceptable.

Social identity theory posits that groups strive to maintain a positive self‐concept, often using social creativity strategies like reframing situations, altering comparisons, and emphasizing group virtues, especially when their status or moral standing is threatened. These strategies are particularly salient in contexts where the group's status or moral standing is threatened. When the actions of a group are perceived as morally questionable or outright unethical by outsiders, maintaining a positive group identity becomes challenging. However, groups may not simply change their behaviour due to a combination of factors, including strong identification with the group's ideological stance, the use of moral disengagement strategies to justify their actions, and the framing of their position as morally defensible within the group's narrative. Importantly, what outsiders view as morally transgressive may not be regarded as such by the group itself. Instead, groups often reinterpret their actions as aligned with higher values or justified by perceived threats, thus reinforcing their moral framework rather than revising their behaviour. It is in these contexts that social creativity intersects with moral disengagement mechanisms. For instance, when pro‐Russian supporters reframe the invasion of Ukraine as a defence of traditional values, they engage in social creativity by shifting the basis of comparison from the aggressive nature of the invasion to the purported moral high ground of cultural preservation. This reframing is not just a matter of presenting the group favourably, but also represents a form of moral justification. Moral justification, as defined in Bandura's framework, is one of the key mechanisms of moral disengagement. Rather than being contradictory, moral justification operates as a cognitive strategy within the broader process of moral disengagement. It involves reframing morally questionable actions as serving a noble purpose, thereby reducing ethical dissonance and allowing individuals or groups to rationalize behaviours that would otherwise conflict with their moral standards. By portraying the invasion as a morally righteous act, these narratives allow group members to disengage from the ethical implications of their support, reducing cognitive dissonance and sustaining a positive moral identity.

Through this lens, we see social creativity and moral disengagement as not merely parallel processes used concurrently, but as deeply interconnected. Social creativity provides the “narrative scaffolding” that supports moral disengagement, enabling groups to justify morally dubious behaviours while maintaining a sense of moral superiority. Moral disengagement strategies, though grounded in cognitive processes, are primarily enacted and sustained through discourse. This perspective aligns with Billig's (1996) notion of thinking as dialogic and argumentative, wherein moral reasoning is intrinsically a social and linguistic practice. The concept of a “narrative scaffold” captures how these strategies are discursively organized, offering a structure for justifying morally contentious actions within a shared social context.

### Study limitations

This study focuses on social creativity and moral disengagement strategies in the Romanian political context, particularly as they pertain to pro‐Russian narratives. While the theoretical frameworks employed are widely applicable, our findings are grounded in the specific geopolitical and cultural dynamics of Romania and Moldova. By situating the study within this context, we highlight its unique contributions to understanding the intersection of identity, morality, and discourse in polarized political environments.

Rather than aiming for generalisability, the value of this study lies in its capacity to use a specific case to generate nuanced insights, along the same lines of work by Billig and Marinho (2017) on Portuguese politics. These findings may open avenues for future research to test the transferability of these dynamics in other geopolitical contexts, but any general observations made here remain exploratory and subject to further empirical corroboration.

The analysis was based on publicly available online sources, such as open online discussion forums, which may not capture the full diversity of pro‐Russian narratives. Additionally, the focus on Romanian texts may overlook important nuances present in narratives communicated in other languages and involving people from other countries equally affected by conflict. Thus, while we argue that our findings offer some valuable insights, we recognize that they do not fully represent the broader landscape of pro‐Russian narratives.

To address these limitations, future research may consider incorporating a broader range of data sources, perhaps including private or less accessible online forums, to investigate whether a wider range of sources might provide insights into additional themes. Expanding the analysis to include texts in other languages, particularly those with key geopolitical relevance, could also provide a more nuanced understanding of the strategies employed. Additionally, using mixed‐method approaches such as combining quantitative content analysis with qualitative interviews or focus groups could strengthen our findings (Creswell & Plano Clark, 2017).

Future research could further explore the discursive dimensions of moral justifications as social practices, building on the findings of this study. Moral reasoning extends beyond cognitive processes, functioning as a social and dialogic activity, as evidenced by the narratives analysed here. Investigating moral justifications as situated discursive actions could enhance understanding of how individuals and groups negotiate accountability and legitimacy in morally contested contexts. For instance, Tileagă's work on discourse and public accountability can provide a robust methodological foundation for examining the construction and contestation of moral and political accountability in interactional contexts (Tileagă, 2010). This framework can guide research on how moral justifications are negotiated in various communicative settings, such as interviews or public debates, by analysing how individuals and groups draw on cultural and ideological narratives to legitimize actions, assign responsibility, or challenge moral norms. Such an approach situates moral reasoning within the dynamic context of discourse, advancing the understanding of morality's cognitive and social dimensions. Overall, this approach could provide greater insight into how moral disengagement strategies are expressed, contested, and maintained in everyday discourse. By examining the interaction between discourse and social identity, future research could further our understanding of the broader implications of these narratives for intergroup relations and political polarization.

### Conclusion

Understanding how pro‐Russian narratives use social creativity and moral disengagement can have significant implications for public opinion, political discourse, and policy decisions. Identifying and recognizing these strategies is important for developing effective counter‐narratives and interventions aimed at mitigating the spread of misinformation and propaganda in situations of violent intergroup conflict. By understanding in more depth the rhetorical and psychological strategies used within pro‐Russian narratives, this study contributes to broader scholarship to counteract the public's vulnerability to misinformation and to develop stronger media literacy.

## AUTHOR CONTRIBUTIONS

**Ana‐Maria Bliuc:** Conceptualization; data curation; formal analysis; writing – original draft; writing – review and editing; methodology. **Daniela Muntele‐Hendreș:** Writing – review and editing; data curation; formal analysis; methodology.

## Data Availability

The data that support the findings of this study are available on request from the corresponding author. The data are not publicly available due to privacy or ethical restrictions.

## References

[bjso12878-bib-0074] Ayal, S. , & Gino, F. (2012). Honest rationales for dishonest behavior. In M. Mikulincer & P. R. Shaver (Eds.), The social psychology of morality: Exploring the causes of good and evil (pp. 149–166). American Psychological Association. 10.1037/13091-008

[bjso12878-bib-0004] Bandura, A. (1990). Selective activation and disengagement of moral control. Journal of Social Issues, 46(1), 27–46. 10.1111/j.1540-4560.1990.tb00270.x

[bjso12878-bib-0005] Bandura, A. (1999). Moral disengagement in the perpetration of inhumanities. Personality and Social Psychology Review, 3(3), 193–209. 10.1207/s15327957pspr0303_3 15661671

[bjso12878-bib-0006] Bandura, A. (2002). Selective moral disengagement in the exercise of moral agency. Journal of Moral Education, 31(2), 101–119. 10.1080/0305724022014322

[bjso12878-bib-0007] Bar‐Tal, D. (1990). Causes and consequences of delegitimization: Models of conflict and ethnocentrism. Journal of Social Issues, 46(1), 65–81. 10.1111/j.1540-4560.1990.tb00277.x

[bjso12878-bib-0008] Bar‐Tal, D. , Oren, N. , & Nets‐Zehngut, R. (2014). Sociopsychological analysis of conflict‐supporting narratives: A general framework. Journal of Peace Research, 51(5), 662–675. 10.1177/0022343314533984

[bjso12878-bib-0009] Bennett, W. L. , & Livingston, S. (2018). The disinformation order: Disruptive communication and the decline of democratic institutions. European Journal of Communication, 33(2), 122–139. 10.1177/0267323118760317

[bjso12878-bib-0082] Billig, M. (1996). Arguing and thinking: A rhetorical approach to social psychology. Cambridge University Press.

[bjso12878-bib-0081] Billig, M. , & Marinho, C. (2017). The politics and rhetoric of commemoration: How the Portuguese parliament celebrates the 1974 revolution. Bloomsbury Publishing.

[bjso12878-bib-0011] Bliuc, A. M. , Betts, J. M. , Vergani, M. , Bouguettaya, A. , & Cristea, M. (2024). A theoretical framework for polarization as the gradual fragmentation of a divided society. Communications Psychology, 2(1), 75. 10.1038/s44170-023-00098-4 39242900 PMC11327288

[bjso12878-bib-0012] Bliuc, A. M. , Bouguettaya, A. , & Felise, K. D. (2021). A theoretical framework for polarization as the gradual fragmentation of a divided society. Communications Psychology, 2(1), 4744. 10.3389/fpsyg.2021.759693 PMC11327288 39242900

[bjso12878-bib-0013] Bliuc, A. M. , & Chidley, A. (2022). From cooperation to conflict: The role of collective narratives in shaping group behaviour. Social and Personality Psychology Compass, 16(7), e12670. 10.1111/spc3.12670

[bjso12878-bib-0014] Bliuc, A. M. , Hamilton, T. , & Muntele, D. (2024). Diversity, dissent, and fragmentation in the #MeToo movement: The role of collective and individual dimensions. Frontiers in Psychology, 15, 1290065. 10.3389/fpsyg.2024.1290065 39035096 PMC11259091

[bjso12878-bib-0015] Bliuc, A. M. , McGarty, C. , Thomas, E. F. , Lala, G. , Berndsen, M. , & Misajon, R. (2015). Public division about climate change rooted in conflicting socio‐political identities. Nature Climate Change, 5(3), 226–229. 10.1038/nclimate2507

[bjso12878-bib-0016] Braun, V. , & Clarke, V. (2006). Using thematic analysis in psychology. Qualitative Research in Psychology, 3(2), 77–101. 10.1191/1478088706qp063oa

[bjso12878-bib-0079] Braun, V. , & Clarke, V. (2019). Reflecting on reflexive thematic analysis. Qualitative Research in Sport, Exercise and Health, 11(4), 589–597. 10.1080/2159676x.2019.1628806

[bjso12878-bib-0017] Brewer, M. B. (1999). The psychology of prejudice: Ingroup love and outgroup hate? Journal of Social Issues, 55(3), 429–444. 10.1111/0022-4537.00126

[bjso12878-bib-0020] Creswell, J. W. , & Plano Clark, V. L. (2017). Designing and conducting mixed methods research (3rd ed.). Sage.

[bjso12878-bib-0021] Douglas, K. M. , McGarty, C. , Bliuc, A. M. , & Lala, G. (2005). Understanding cyberhate: Social competition and social creativity in online white supremacist groups. Social Science Computer Review, 23(1), 68–76. 10.1177/0894439304271538

[bjso12878-bib-0080] Douglas, K. M. , Uscinski, J. E. , Sutton, R. M. , Cichocka, A. , Nefes, T. , Ang, C. S. , & Deravi, F. (2019). Understanding conspiracy theories. Political Psychology, 40(S1), 3–35. 10.1111/pops.12568

[bjso12878-bib-0022] Dunning, D. (2007). Self‐image motives and consumer behavior: How sacrosanct self‐beliefs sway preferences in the marketplace. Journal of Consumer Psychology, 17(4), 237–249. 10.1016/S1057-7408(07)70033-5

[bjso12878-bib-0024] Geissler, D. , Bär, D. , Pröllochs, N. , & Feuerriegel, S. (2022). Russian propaganda on social media during the 2022 invasion of Ukraine. EPJ Data Science, 12, 1–20. 10.1140/epjds/s13688-023-00414-5

[bjso12878-bib-0025] Gini, G. , Pozzoli, T. , & Bussey, K. (2015). Collective moral disengagement: Initial validation of a scale for adolescents. European Journal of Developmental Psychology, 12(5), 462–474. 10.1080/17405629.2015.1039304

[bjso12878-bib-0076] Gollwitzer, M. , & Melzer, A. (2012). Macbeth and the joystick: Evidence for moral cleansing after playing a violent video game. Journal of Experimental Social Psychology, 48(6), 1356–1360. 10.1016/j.jesp.2012.07.001

[bjso12878-bib-0028] Halperin, E. , & Gross, J. J. (2011). Emotion regulation in violent conflict: Revisiting the view from the inside. Current Directions in Psychological Science, 20(6), 409–413. 10.1177/0963721411422069

[bjso12878-bib-0029] Hammack, P. L. (2008). Narrative and the cultural psychology of identity. Personality and Social Psychology Review, 12(3), 222–247. 10.1177/1088868308316892 18469303

[bjso12878-bib-0030] Hammack, P. L. (2011). Narrative and the politics of identity: The cultural psychology of Israeli and Palestinian youth. Oxford University Press. 10.1093/acprof:oso/9780195394467.001.0001

[bjso12878-bib-0031] Haslam, N. (2006). Dehumanization: An integrative review. Personality and Social Psychology Review, 10(3), 252–264. 10.1207/s15327957pspr1003_4 16859440

[bjso12878-bib-0032] Haslam, S. A. (2001). Psychology in organizations: The social identity approach. Sage.

[bjso12878-bib-0033] Hilton, D. , & Liu, J. (2017). History as the narrative of a people: From function to structure and content. Memory Studies, 10, 297–309. 10.1177/1750698017701612

[bjso12878-bib-0034] Hogg, M. A. (2016). Social identity theory. In Understanding peace and conflict through social identity theory (pp. 3–17). Springer. 10.1007/978-3-319-29869-6_1

[bjso12878-bib-0077] Huang, G. , & Yan, M. N. (2014). Why groups engage in collective deviance? The role of unethical leadership. Academy of Management Proceedings, 2014(1), 13365. 10.5465/ambpp.2014.13365abstract

[bjso12878-bib-0037] Jetten, J. , Schmitt, M. T. , Branscombe, N. R. , & McKimmie, B. M. (2005). Suppressing the negative effect of devaluation on group identification: The role of intergroup differentiation and intragroup respect. Journal of Experimental Social Psychology, 41(2), 208–215. 10.1016/j.jesp.2004.07.012

[bjso12878-bib-0078] Pozzoli, T. , Gini, G. , & Vieno, A. (2012). Individual and class moral disengagement in bullying among elementary school children. Aggressive Behavior, 38(5), 378–388. 10.1002/ab.21442 22778018

[bjso12878-bib-0040] Khomyakov, M. (2023). Thinking of war, facing the catastrophe: The Russian‐Ukrainian war. European Journal of Social Theory, 26(4), 514–526. 10.1177/13684310231172599

[bjso12878-bib-0043] Liu, J. H. , & Hilton, D. (2005). How the past weighs on the present: Social representations of history and their role in identity politics. British Journal of Social Psychology, 44(4), 537–556. 10.1348/014466605X27162 16368018

[bjso12878-bib-0044] Liu, J. H. , Sibley, C. G. , & Huang, L. L. (2014). History matters: The impact of culture‐specific symbols on political attitudes and intergroup relations. Political Psychology, 35(1), 57–79. 10.1111/pops.12040

[bjso12878-bib-0045] Livingstone, A. G. , & Haslam, S. A. (2007). The importance of social identity content in a setting of chronic social conflict: Understanding intergroup relations in Northern Ireland. British Journal of Social Psychology, 46(1), 1–23. 10.1348/014466605X85723 17535458

[bjso12878-bib-0046] McAlister, A. L. , Bandura, A. , & Owen, S. V. (2006). Mechanisms of moral disengagement in support of military force: The impact of sept. 11. Journal of Social and Clinical Psychology, 25(2), 141–165. 10.1521/jscp.2006.25.2.141

[bjso12878-bib-0073] McLaughlin, B. (2020). Tales of conflict: Narrative immersion and political aggression in the United States. Media Psychology, 23(4), 579–602. 10.1080/15213269.2019.1611452

[bjso12878-bib-0048] Newman, C. M. , Le, H. N. , North‐Samardzic, A. , & Cohen, M. (2019). Group moral disengagement: Scale development and cross‐cultural validation. Journal of Business Ethics, 156(1), 183–198. 10.1007/s10551-017-3540-6

[bjso12878-bib-0052] Pehrson, S. , Brown, R. , & Zagefka, H. (2009). When does national identification lead to the rejection of immigrants? Cross‐sectional and longitudinal evidence for the role of essentialist in‐group definitions. British Journal of Social Psychology, 48(1), 61–76. 10.1348/014466608X288827 18302807

[bjso12878-bib-0053] Reicher, S. , Spears, R. , & Haslam, S. A. (2006). The social identity approach in social psychology. In M. Hogg & J. Cooper (Eds.), The Sage handbook of social psychology (pp. 179–194). Sage. 10.4135/9781848608221.n10

[bjso12878-bib-0054] Reicher, S. D. , Cassidy, C. , Wolpert, I. , Hopkins, N. , & Levine, M. (2006). Saving Bulgaria's Jews: An analysis of social identity and the mobilisation of social solidarity. European Journal of Social Psychology, 36(1), 49–72. 10.1002/ejsp.291

[bjso12878-bib-0055] Reicher, S. D. , Haslam, S. A. , Spears, R. , & Reynolds, K. J. (2012). A social mind: The context of John Turner's work and its influence. European Review of Social Psychology, 23(1), 344–385. 10.1080/10463283.2012.745672

[bjso12878-bib-0056] Sachdeva, S. , Iliev, R. , & Medin, D. L. (2009). Sinning saints and saintly sinners: The paradox of moral self‐regulation. Psychological Science, 20(4), 523–528. 10.1111/j.1467-9280.2009.02326.x 19320857

[bjso12878-bib-0075] Shalvi, S. , Gino, F. , Barkan, R. , & Ayal, S. (2015). Self‐serving justifications. Current Directions in Psychological Science, 24(2), 125–130. 10.1177/0963721414553264

[bjso12878-bib-0060] Sokolova, A. (2023). Opponents and proponents of the war in Ukraine in Russian social media: Who are they? ArXiv 10.48550/arXiv.2308.04473

[bjso12878-bib-0061] Tajfel, H. , & Turner, J. C. (1979). An integrative theory of intergroup conflict. In W. G. Austin & S. Worchel (Eds.), The social psychology of intergroup relations (pp. 33–47). Brooks/Cole.

[bjso12878-bib-0062] Tileagă, C. (2010). Political accountability, public constitution of recent past and the collective memory of socio‐political events: A discursive analysis. Journal of Community & Applied Social Psychology, 20(5), 363–376. 10.1002/casp.1043

[bjso12878-bib-0063] Tsang, J.‐A. (2002). Moral rationalization and the integration of situational factors and psychological processes in immoral behavior. Review of General Psychology, 6(1), 25–50. 10.1037/1089-2680.6.1.25

[bjso12878-bib-0064] Turner, J. C. , Hogg, M. A. , Oakes, P. J. , Reicher, S. D. , & Wetherell, M. S. (1987). Rediscovering the social group: A self‐categorization theory. Basil Blackwell.

[bjso12878-bib-0065] Van Bezouw, M. J. , van der Toorn, J. , & Becker, J. C. (2021). Social creativity: Reviving a social identity approach to social stability. European Journal of Social Psychology, 51(2), 409–422. 10.1002/ejsp.2746

[bjso12878-bib-0066] Van Leeuwen, F. , & Park, J. H. (2009). Perceptions of social dangers, moral foundations, and political orientation. Personality and Individual Differences, 47(3), 169–173. 10.1016/j.paid.2009.02.017

[bjso12878-bib-0068] Waisbord, S. (2018). Truth is what happens to news: On journalism, fake news, and post‐truth. Journalism Studies, 19(13), 1866–1878. 10.1080/1461670X.2018.1492881

[bjso12878-bib-0070] Wenzel, M. , Mummendey, A. , & Waldzus, S. (2007). Superordinate identities and intergroup conflict: The ingroup projection model. European Review of Social Psychology, 18(1), 331–372. 10.1080/10463280701728302

[bjso12878-bib-0071] Wenzel, M. , Waldzus, S. , & Steffens, M. (2016). Ingroup projection as a challenge of diversity: Consensus about and complexity of superordinate categories. In J. F. Dovidio , M. Hewstone , P. Glick , & V. M. Esses (Eds.), The SAGE handbook of prejudice, stereotyping and discrimination (pp. 82–101). Cambridge University Press. 10.1017/9781316161579.004

